# Making urinary extracellular vesicles a clinically tractable source of biomarkers for inherited tubulopathies using a small volume precipitation method: proof of concept

**DOI:** 10.1007/s40620-019-00653-8

**Published:** 2019-10-04

**Authors:** Timothy Lee Williams, Carlos Bastos, Nuno Faria, Fiona Eve Karet Frankl

**Affiliations:** 1 Department of Veterinary Medicine, University of Cambridge, Madingley Road, Cambridge, CB3 0ES UK; 2 Department of Medical Genetics and Division of Renal Medicine, University of Cambridge and Cambridge University Hospitals Foundation Trust, Cambridge, UK

**Keywords:** Gitelman Syndrome, Precipitation, Exosome

## Abstract

Biomarkers of inherited tubulopathies would be useful for clarifying diagnoses in patients where genetic screening is not readily available or where disease-attributable mutations are not found. Urinary extracellular vesicles (uEVs) obtained by ultracentrifugation can be used as a source of biomarkers for inherited tubulopathies such as Gitelman Syndrome (GS), however, ultracentrifugation requires costly equipment and is thus not usually accessible. In contrast, precipitation methods can extract uEVs using standard laboratory centrifuges, thus making uEVs extracted by this method clinically tractable as a source of biomarkers for GS and other inherited tubulopathies. Here we optimise a precipitation method for extracting urinary extracellular vesicles (uEVs) and provide proof of concept that these uEVs are a source of biomarkers using GS an exemplar tubulopathy. For method optimisation, uEVs were precipitated from fresh and frozen (for up to 6 years), small volume (1–2 mL) urine samples from healthy volunteers and GS patients. Nanoparticle tracking analysis was used to calculate the concentration of uEVs. Thiazide sensitive sodium-chloride cotransporter (NCC) content was determined by densitometry of Western blots. NCC content of uEVs was lower in GS patients (n = 11) than healthy volunteers (n = 12; P = 0.001). Three of four patients clinically suspected for GS, in whom only a single *SLC12A3* mutation was identified, had lower uEV NCC content than all healthy volunteers tested. In the clinical setting, sufficient uEVs can be extracted from frozen, small volume urine samples using precipitation methods to distinguish patients with GS from healthy volunteers, and thus this source of uEVs could be utilised as an additional diagnostic test for GS and similar disorders.

**Electronic supplementary material:**

The online version of this article (10.1007/s40620-019-00653-8) contains supplementary material, which is available to authorized users.

## Introduction

Inherited tubulopathies are rare transporter disorders of renal tubular epithelia causing defective renal handling of electrolytes, amino-acids, water and/or glucose. In Gitelman Syndrome (GS; loss of thiazide-sensitive sodium-chloride cotransporter function), genetic testing has a reported clinical sensitivity of only 65–80% [1], therefore identification of biomarkers of inherited tubulopathies (including GS) would be useful in patients where there is clinical doubt and/or where genetic testing is not readily available.

Urinary extracellular vesicles (uEVs) are a potentially rich source of biomarkers for tubulopathies, since their membranes are composed of apical proteins from all nephron segments [2]. Thiazide-sensitive sodium-chloride cotransporter (NCC) abundance within uEVs of GS patients obtained by ultracentrifugation is reduced on Western blot [3, 4]; however, ultracentrifugation requires costly equipment, and so this method of uEV extraction would not be easily accessible to clinicians for diagnostic purposes. Commercially available kits allow extraction of uEVs by precipitation using standard laboratory centrifuges; this might make the use of uEVs as a source of biomarkers more widely applicable and clinically tractable. Here we provide proof of concept that uEVs extracted by precipitation from small-volume urine samples can be so used, using GS as an exemplar tubulopathy. Therefore, this technique could be adapted to yield a clinically tractable additional diagnostic test for GS and potentially other tubulopathies.

## Materials and methods

The Exiqon miRCURY Exosome Isolation Kit (#300102) was used according to manufacturer protocol for extraction of uEVs, with the addition of a filtration step (0.22 µm filter, Millipore Stericup 250) after harvesting of the initial supernatant, and resuspension of the final pellet containing uEVs in 50 µL of resuspension buffer (rather than 100 µL). We evaluated a modification to the standard protocol with the aim of increasing uEV yield; fresh urine samples without dipstick abnormalities (Siemens Multistix 10SG) were collected from healthy volunteers into sterilised glass bottles and incubated with precipitation buffer, either overnight or for the manufacturer-recommended time of 60 min, at 4 °C. Total protein concentrations of uEV preparations were measured using a bioinchoninic acid (BCA) method (Pierce™ BCA Protein Assay Kit, Thermo Scientific), and particle numbers determined by nanoparticle tracking analysis on a Nanosight NS500 (Supplementary Figure 1, Malvern, UK). Comparisons between 60 min and overnight incubation, and precipitation and ultracentrifugation were made using the Wilcoxon signed-rank test; data are presented as median [range].

To provide proof of concept that uEVs could be used as a source of biomarkers in inherited tubulopathies, azide-preserved small volume (1–2 mL) urine samples from healthy volunteers and patients with GS, stored at − 80 °C, were obtained from the sample bank of the Cambridge Renal Genetic and Tubular Disorders service. Samples were collected under Cambridgeshire Research Ethics Committee approval (08-H0306-62) with informed consent of participants. Protease inhibitors (Roche cOmplete, EDTA-free) were added at the point of thawing. Since cooling increases uromodulin polymerisation [5], samples were alkalinised by addition of 1 M NaOH to pH 8.1 (determined using MColorpHast™ pH indicator strips) prior to processing, to reverse this tendency [6] and maximise uEV yield.

To exclude an adverse effect of alkalinisation on protein and uEVs extraction by precipitation, further fresh urine samples were collected from healthy volunteers (n = 9) and divided into two aliquots, one of which was alkalinised (as per protocol above) prior to processing. Protein and particle yields were compared using the Wilcoxon signed rank test.

## Results

Overnight incubation of samples with precipitation buffer significantly increased particle concentration within uEV preparations (1.6 × 10^10^/mL [0.3–3.5 × 10^10^/mL] vs. 0.07 × 10^10^/mL [0.03–0.2 × 10^10^/mL]; P = 0.03), hence overnight incubation was performed thereafter. Total protein content of uEV preparations was unchanged (3.8 [3.1–5.1] vs. 3.3 [1.8–6.4] µg/mL; n = 6, P = 0.3). The presence of uEVs within preparations obtained by precipitation was confirmed by Western blotting for CD9 and TSG101 (Supplementary Figure 2).

The precipitation method yielded a similar quantity of protein and uEVs (on a per mL urine basis) compared to ultracentrifugation (as per our previously published protocol [7]); protein contents were 0.7–10.8 µg (0.7–10.8 µg/mL urine) and 12.1–50.8 µg (0.2–0.8 µg/mL urine) in uEV preparations produced by precipitation and ultracentrifugation respectively (n = 3; P = 0.2). Total number of uEVs from these preparations were 6.2–28.0 billion and 1.0–27.8 billion particles/mL urine respectively (n = 3; P = 0.4).

Protein content of uEV preparations obtained from frozen samples (used as surrogate marker of uEV quantity) ranged from 1.2 to 30.9 µg. Thirty microlitre aliquots of uEV preparations were reduced and separated by 4–12% SDS-PAGE (NuPAGE, Thermo Fisher) prior to transfer to nitrocellulose membranes (Bio-Rad). Membranes were blocked with 5% skimmed milk in 0.1% PBS-Tween 20, and incubated with primary (anti-NCC [Abcam, ab95032], anti-CD9 [Abcam, ab92729] or anti-TSG101 [Abcam, ab83]) and secondary (IRDye^®^ or HRP-conjungated) antibodies (Dako). Bands of appropriate size were observed using an infrared imaging system (LICOR Odyssey) or on radiographic film (following addition of chromographic reagent), and densitometry performed using ImageJ software (NIH, USA). Comparisons between groups were made using the Mann–Whitney U test.

**Fig. 1:**
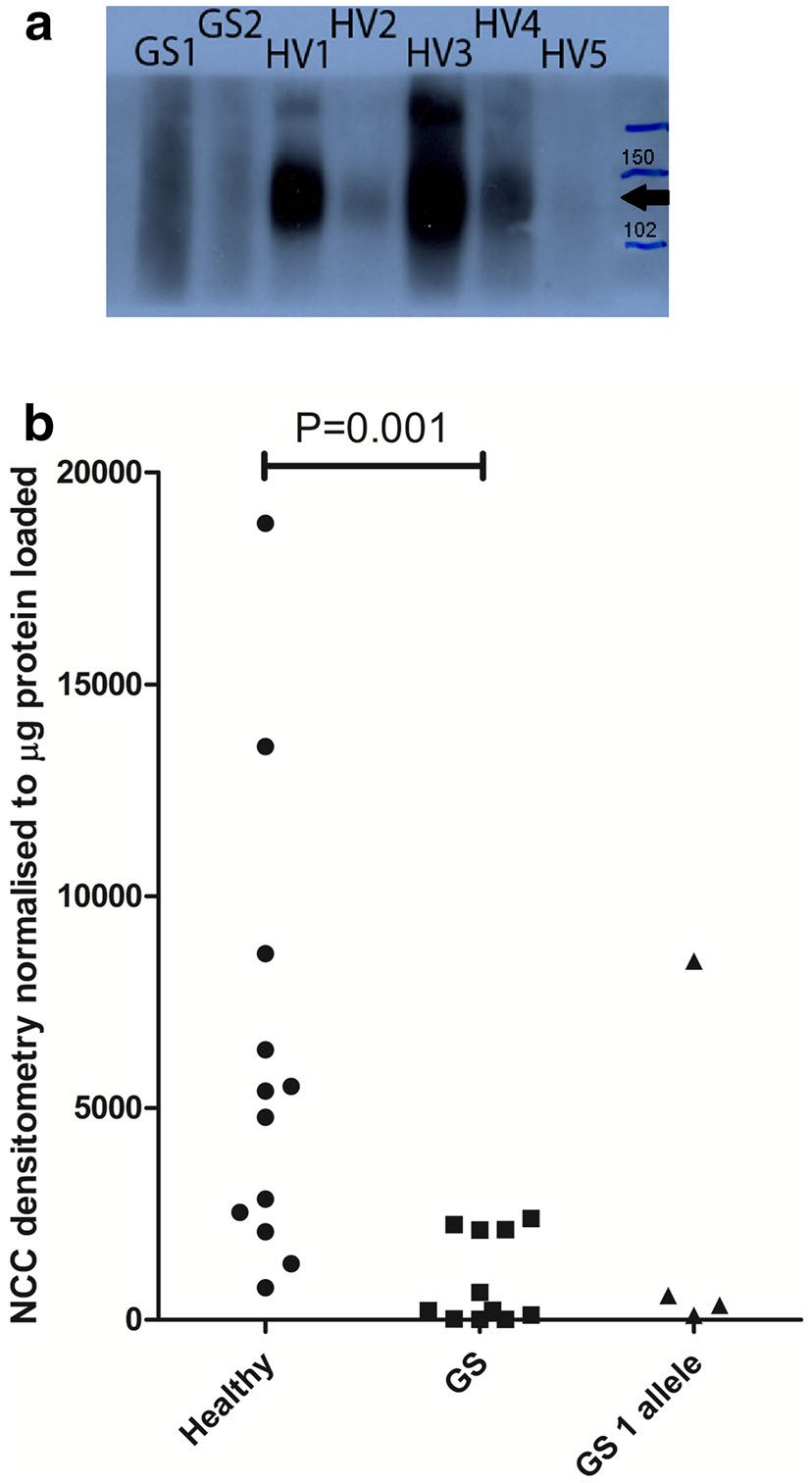
**a** Representative western blot of urinary extracellular vesicle (uEV) preparations, obtained from small volume urine samples from healthy volunteers (HV) and patients with genetically confirmed Gitelman Syndrome (GS), for the thiazide sensitive sodium-chloride cotransporter (NCC). Expected size is ~ 111 kDa (indicated by the black arrow). Molecular size markers (in kDa) are shown in the right hand lane. uEVs were extracted from 1 to 2 mL urine samples, previously stored at − 80 °C, by precipitation. **b** Scatter graph showing densitometry results from Western blot for the thiazide sensitive sodium-chloride cotransporter (NCC) in urinary extracellular vesicles (uEVs) obtained from small volume urine samples from healthy volunteers, patients with confirmed Gitelman Syndrome (GS) based on genetic testing, and patients clinically suspected to have GS for which only one known mutation of *SLC12A3* could be identified (GS 1 allele). Densitometry readings for NCC were normalised to the amount of protein loaded (in µg). uEV NCC content was significantly lower in GS patients compared to healthy volunteers (*P* = 0.001)

NCC content of uEVs, normalised to quantity of protein loaded, was significantly lower in GS patients (n = 11) than healthy volunteers (n = 12; P = 0.001, Fig. 1). Three of four patients clinically suspected to have GS, but in whom only a single *SLC12A3* mutation was identified, also had lower uEV NCC content than all healthy volunteers tested (Fig. 1).

Alkalinisation did not adversely affect either protein concentration (non-alkalinised 3.9 µg/mL [1.0–6.6 µg/mL], alkalinised 4.8 µg/mL [0.9–9.1 µg/mL]; n = 9; P = 0.25) or particle yield (non-alkalinised 2.3x10^10^ [2.4 × 10^9^ – 4.2 × 10^10^] particles/mL, alkalinised 1.4 × 10^10^ [5.3 × 10^8^ – 3.2 × 10^10^] particles/mL; P = 0.13).

## Discussion

This study demonstrates that sufficient uEVs can be extracted from small volume urine samples by precipitation methods for immunoblotting studies. To the authors’ knowledge, this is the first study to demonstrate that the quantity of NCC present within uEVs extracted by precipitation is lower in patients with GS than in healthy volunteers. Furthermore, the abundance of NCC within uEVs of most patients suspected of GS but without a confirmed genetic diagnosis was also low, thus indicating that this method could be a useful additional test for GS in patients in which a diagnosis is not reached by genetic testing. These findings concur with those of a previous study which evaluated uEVs extracted from GS patients by ultracentrifugation [3]. Further studies to compare the uEVs extracted using both techniques would be of potential interest, but clinicians are unlikely to have routine access to ultracentrifuges, so that technique is not generalizable. The main advantage of the precipitation technique validated here is that extraction of uEVs can be performed using standard laboratory centrifuges, thus making uEVs a widely accessible and clinically tractable source of biomarkers for GS and other inherited tubulopathies. These are encouraging data, and further refinement and investigation of this technique as a diagnostic test for GS is warranted.

Normalised NCC abundance within uEV preparations of some GS patients was similar to healthy volunteers, perhaps reflecting expression of some mutant NCC proteins at the apical membrane, and thus within uEVs [8]. It is recognised that suitable normalisation of uEVs is problematic because no single parameter can specifically quantify EVs; and EV subtypes from different nephron segments will express different markers [9]. We used total protein for this purpose because it is a clinically accessible surrogate marker for all uEVs, and our aim was to demonstrate proof of concept for a clinically tractable test. Although we acknowledge that total protein is a non-specific surrogate marker of uEV number, we would have expected that using it would if anything decrease the likelihood of identifying a significant difference between the normalised NCC content of healthy volunteers and GS patients as in the present study. The main limitation of our study was the relatively small number of GS patient samples included in the proof of concept study, which reflects the ultra-rarity of GS in the population (predicted prevalence approximately 1:40,000 [1]). However, our study was still adequately powered to demonstrate a significant difference in the NCC content of uEVs between GS patients and healthy volunteers.

Our technique could also improve characterisation of the molecular bases of other inherited renal tubulopathies. For example, excretion of aquaporin-2 (AQP2) within uEVs correlates with AQP2 expression in the kidneys of humans and rats [4, 10], suggesting that the uEV proteome is a surrogate marker for expression of apical proteins within the nephron. That said, hydration status affects AQP2 expression, so even this protein would not be reliable as a denominator of EV amount in healthy volunteers. Excretion of various proteins within uEVs of humans and rats has been reported [4, 10], and the precipitation techniques utilised here will allow future studies of the uEV proteome to be performed using urine samples of lower volume. This could be also particularly beneficial when studying animal models in which urine volumes are low.

## Electronic supplementary material

Below is the link to the electronic supplementary material.

Supplementary material 1 (DOCX 314 kb)

## References

[1] Blanchard A (2017). Gitelman syndrome: consensus and guidance from a Kidney Disease: Improving Global Outcomes (KDIGO) Controversies Conference. Kidney Int.

[2] De Palma G, Sallustio F, Schena FP (2016). Clinical application of human urinary extracellular vesicles in kidney and urologic diseases. Int J Mol Sci.

[3] Corbetta S (2015). Urinary exosomes in the diagnosis of Gitelman and Bartter syndromes. Nephrol Dial Transplant.

[4] Joo KW (2007). Reduced urinary excretion of thiazide-sensitive Na-Cl cotransporter in Gitelman syndrome: preliminary data. Am J Kidney Dis.

[5] Fernandez-Llama P (2010). Tamm-Horsfall protein and urinary exosome isolation. Kidney Int.

[6] Kobayashi K, Fukuoka S (2001). Conditions for solubilization of Tamm-Horsfall protein/uromodulin in human urine and establishment of a sensitive and accurate enzyme-linked immunosorbent assay (ELISA) method. Arch Biochem Biophys.

[7] Gracia T (2017). Urinary exosomes contain MicroRNAs capable of paracrine modulation of tubular transporters in kidney. Sci Rep.

[8] De Jong JC (2002). Functional expression of mutations in the human NaCl cotransporter: evidence for impaired routing mechanisms in Gitelman’s syndrome. J Am Soc Nephrol.

[9] Thery C (2018). Minimal information for studies of extracellular vesicles 2018 (MISEV2018): a position statement of the International Society for Extracellular Vesicles and update of the MISEV2014 guidelines. J Extracell Vesicles.

[10] Wen H (1999). Urinary excretion of aquaporin-2 in rat is mediated by a vasopressin-dependent apical pathway. J Am Soc Nephrol.

