# Supplementary material for: Making urinary extracellular vesicles a clinically tractable source of biomarkers for inherited tubulopathies using a small volume precipitation method: proof of concept
Source: J Nephrol. 2019 Oct 4;33(2):383–6. doi: 10.1007/s40620-019-00653-8 (PMC7118034; doi:10.1007/s40620-019-00653-8)

**Supplementary Figure 1.** Size of microvesicles obtained from a urinary extracellular vesicle (uEV) preparation (1:200 dilution); urine was processed using a commercially available kit that utilises volume-excluding polymers to enhance uEV recovery.

Numbers represent the size of particles (in nm) corresponding to the peaks on the graph.


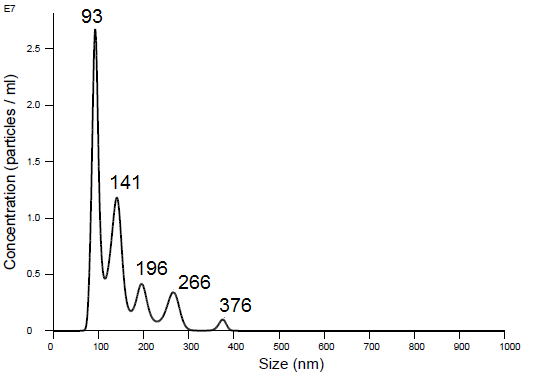


**Supplementary Figure 2**. Western blots for the canonical extracellular vesicle markers CD9 and TSG101, which confirmed the presence of EVs within a subset of urinary extracellular vesicle (uEV) preparations obtained by precipitation following incubation with Precipitation Buffer B for 60 minutes and overnight (approximately 16-18 hours). Expected sizes of CD9 and TSG101 are ~25kDa and ~50kDa respectively. Due to small volume and low protein content of uEV preparations, only a single western blot could be made from each uEV preparation. The nitrocellulose membrane was cut at 31-38kDa to enable two antibodies to be used.

The uEV preparations were extracted from individual urine samples (A, B, C) from healthy volunteers using the same precipitation kit as in Figure S1. Two uEV preparations were obtained from each sample; one 10mL aliquot of urine was incubated with Precipitation Buffer B from the kit for 60 minutes at 4^o^C (60m), whereas the second 10mL aliquot of the same urine sample was incubated with Precipitation Buffer B overnight (approximately 16-18 hours) at 4^o^C (O/N).


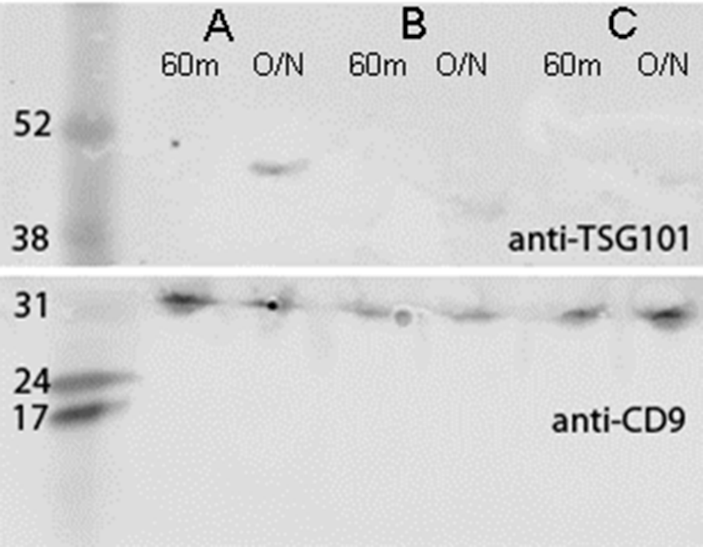

Supplement: Supplementary file 1 — Supplementary material 1 (DOCX 314 kb) [file 40620_2019_653_MOESM1_ESM.docx]
